# Phenotype response for the invasive *Petaurus notatus* in Tasmania

**DOI:** 10.1093/jmammal/gyaf074

**Published:** 2025-10-27

**Authors:** Meagan Powley, Katarina Mikac

**Affiliations:** School of Science, Environmental Futures, University of Wollongong, Northfields Ave, Wollongong, NSW 2500, Australia; School of Science, Environmental Futures, University of Wollongong, Northfields Ave, Wollongong, NSW 2500, Australia

**Keywords:** dietary switch, geometric morphometrics, glider, *Petauridae*, phenotype response

## Abstract

A mainland native nocturnal arboreal glider *Petaurus notatus* (Krefft’s Glider) was introduced to Tasmania in the 1830s as a pet and its subsequent escape and landscape spread has been associated with the predation of endemic and migratory birds. Using geometric morphometrics, we assessed whether *P. notatus* experienced a phenotypic response, visible in their skull, after introduction to Tasmania. We found significant skull form variation between Tasmanian (*n *= 57) and Victorian (*n *= 102) glider specimens. Specifically, there is an increase in rostrum length—particularly at the mid-cranium—a dorsal shift of the frontal bones, and increase in posterior angle of the coronoid process on the mandible. Whether this morphological change is associated with a dietary shift towards carnivory or because of multiple ecological pressures is unclear. However, similar morphological variations identified in the Tasmanian specimens have previously been linked to an increase in carnivory in other species as noted by other researchers.

## Teaser

*Petaurus notatus* (Krefft’s Glider), introduced to Tasmania in the 1830s, has been linked to bird predation. Using geometric morphometrics, we found that Tasmanian gliders have larger skulls with features linked to carnivory.

During a peak period in Australian history between 1836 and 1880, over 60 species of vertebrates were released into the Australian environment. Many of the mammalian species remain a serious contemporary Australian environmental problem, including *Sus scrofa* (Eurasian Wild Pig), *Capra hircus* (Domestic Goat), *Oryctolagus cuniculus* (European Rabbit), various species of deer, *Felis catus* (Domestic Cat), *Vulpes vulpes* (Red Fox), and others (Redhead et al. 1991; Krull et al. 2014; Kinnear 2018). The introduction of these species by Europeans was primarily for agricultural, aesthetics, and entertainment purposes such as game hunting (Redhead et al. 1991). While successful establishment of an introduced non-native species to a novel environment is generally low (Williamson 1996; Clout and Russell 2008; Westley 2011; Lockwood et al. 2013), those introduced to a climatically suitable, large geographical area that is predator free are more successful (Forsyth et al. 2004; Clout and Russell 2008), especially when introductions are repeated many times, e.g., red foxes (Fairfax 2019). When species are translocated outside their native range, they can incite biological changes often associated with negative effects on the new biotic community (Short and Smith 1994; Abbott 2006; Kumschick et al. 2015). Often introduced species outcompete native species for dietary and habitat resources. There is evidence for translocated species requiring a dietary shift for success in their establishment phase resulting in an associated shift in skull phenotype (Sakai et al. 2001). When invasive species are introduced to novel locations, previous constraints are lifted (e.g., diet in their native range) that can lead to rapid microevolutionary changes that materialize behaviorally, morphologically, and physiologically (Sakai et al. 2001; Westley 2011; Van Kleeck et al. 2015).

During the peak period of invasive species translocation to Australia, some native animals were translocated as pets to non-endemic regions. An early record describing the introduction of *Petaurus notatus* (Peters 1859), known then as *Petaurus sciureus* and now by the common name Krefft’s Glider (Cremona et al. 2021; Goldingay et al. 2023) to Tasmania noted the physically appealing qualities of the species and anticipated its acceptance as a Tasmanian species (Gunn 1846). The author reported that after many individuals were brought by settlers as pets between 1834 and 1839, they almost immediately escaped from Launceston, and their subsequent invasion of the island was rapid and extensive. Early reports claimed that there was an emerging and concerning trend of predation by domestic cats on the gliders—a predator associated with the destruction of many native Australian species today (Fleming et al. 2022). However, by 1851, the gliders were considered established and were expected to increase in numbers throughout the entire island of Tasmania (Gunn 1851; Lord 1919). Contemporary research supports the forecasted historical accounts, identifying extensive areas of forests occupied by *P. notatus* (Allen et al. 2018); however, rather than several importations of gliders, a recent genetic study showed that it was likely a single event (Campbell et al. 2018).

Mainland *P. notatus* has a seasonally driven omnivorous diet and is an exudate specialist (Smith 1982; Suckling 1984; Howard 1989). However, predation of bird species by Tasmanian *P. notatus* has been reported (Munks et al. 2004; Stojanovic et al. 2014; Heinsohn et al. 2015; Hingston 2019). The characteristics of the predation behavior includes killing and consuming females, juveniles, and the eggs of hollow nesting birds (Stojanovic et al. 2014). This dietary behavior is not limited to Tasmania for *P. notatus*, having been observed on mainland Australia (Fleay 1947; McKenzie et al. 1977; Guppy et al. 2017; Crates et al. 2023), and has also been observed in *P. norfolcensis*, a congener with a similar diet (Holland 2001; Dobson et al. 2005) and distribution overlap (Pavlova et al. 2010). The outcome of this feeding behavior has been linked to a reduction in population sizes of several critically endangered birds in Tasmania (Stojanovic et al. 2014; Heinsohn et al. 2015). Although the diet of *P. notatus* in Tasmania and mainland Australia does include birds, there is evidence to suggest that their behavior may have changed in Tasmania. A recent study found that Tasmanian *P. notatus* were unresponsive to a widely used mainland Australian herbivore bait and preferred a fish-based bait (Owens et al. 2024). Whether this outcome was a regional result for Tasmania or an improved lure more broadly applicable on mainland Australia is untested and not reported in the literature.

Omnivorous invasive species adjust to novel diets more readily than either carnivorous or herbivorous species (Chubaty et al. 2014). For example, the Common Brushtail Possum *Trichosurus vulpecula* is an omnivorous invasive species introduced to New Zealand between 1858 and 1900 (Clout 2000) and experienced a dietary shift to endemic New Zealand floral species. Varieties of New Zealand native trees have been recorded to comprise over 65% of the possum’s diet, and in particular *Weinmannia racemosa*, a floral species now reported to be in decline due to over-browsing by possums in some regions (Campbell 1990; Allen et al. 1997; Cochrane et al. 2003; Husheer 2006). *Trichosurus vulpecula* will also consume the eggs, juveniles, and adults of the native New Zealand birds *Callaeas cinerea wilsoni* (Kokako) and *Apteryx ausaali* (Brown Kiwi; Brown et al. 1993), and the endangered *Wainuia clarki* (Giant Land Snail) as a part of their omnivorous diet (Lawton et al. 2010). Predatory behaviors on birds and scavenging on carcasses by *T. vulpecula* have also been observed in Australia (Rogers et al. 2023; Finnerty et al. 2024). There are no known predators for *T. vulpecula* in New Zealand. Translocated species respond to a change in both biotic and abiotic contexts under extreme evolutionary pressure to survive. As a result, natural processes of evolutionary change can be accelerated and behavioral and morphological modifications are observable more quickly than in their native range (Sakai et al. 2001).

We examined *P. notatus* in their native range in Victoria and introduced range in Tasmania. Our aim was to examine the form of the skull (cranium and mandible) in each region to identify, if present, morphological variation. We predicted that a change in diets would result in changes in cranial and mandibular shape. To test this prediction, cranium (3D laser scans) and mandible (2D photographs) of specimens were analyzed for shape variation using geometric morphometrics methods.

## Methods

### Samples

The material for this research was sourced from all available museum collections in Australia (Appendix S1). A total of 159 available skulls were investigated from: Queen Victoria Museum and Art Gallery (Launceston, *n *= 34); Tasmanian Museum and Art Gallery (Hobart, *n *= 23); Museums Victoria (Melbourne, *n *= 101); and South Australia Museum (Adelaide, *n *= 1). Specimens were entered into museum collections from 1960 to present, apart from 2 specimens from 1915. Adult specimens of *P. notatus* were used and were determined to be adults by the eruption of permanent molars (Cremona et al. 2021). Specimens were attributed to geographic location and sex based on a previously published study and museum records (Cremona et al. 2021). Spatial variables associated with the specimens were collected as unprojected latitude and longitude coordinates and used to determine the origin of each specimen. Specimens were allocated to either Tasmania (female *n *= 30, male *n *= 29) or Victoria (female *n *= 54, male *n *= 48), and regions within Australian state boundaries.

### Data acquisition.

#### Equipment and software.

A SOL PRO 3D Scanner (Scan Dimension, Global Scanning Denmark) was used to acquire all 3D models due to its suitability for scanning objects from 20-170 mm in size and its appropriateness for the size of the specimens. Maximum accuracy is up to 0.05 mm, point distance >0.26 mm, camera resolution 8MP, providing texture color in HDR quality. All scans were taken in a standard anatomical position using a 360° scanning pass with a “High Accuracy” setting. Each specimen was 360° scanned by placing the specimen in the center of the revolving plate of the SOL Pro Scanner with the upper teeth on the turntable surface. Scans were taken in the near position lens setting. Setup requirements for the scanner included the use of a blackout tent following the manufacturer’s instructions, acquiring scans using SOL PRO Creator 2.0.0 software, and aligning scans using SOL PRO Viewer 2.0.0. The final model produced using this technique was a 3D aligned and meshed object suitable for landmark digitizing.

Fixed landmarks were manually applied to the 3D models, guided by texture layers using Slicer V5.4.0 (Fedorov et al. 2012). Multiple photographs of individual specimens contributed to the clarification of anatomical features on 3D models to confirm landmark placement. This program is open-source software developed for clinical and biomedical applications. 2D images used for analysis were obtained using standardized image acquisition techniques (Cardini et al. 2022) and landmarks applied using ImageJ v1.54g (Schneider et al. 2012), an open source program for processing images.

#### Coordinate data points.

**Fig. 1.**
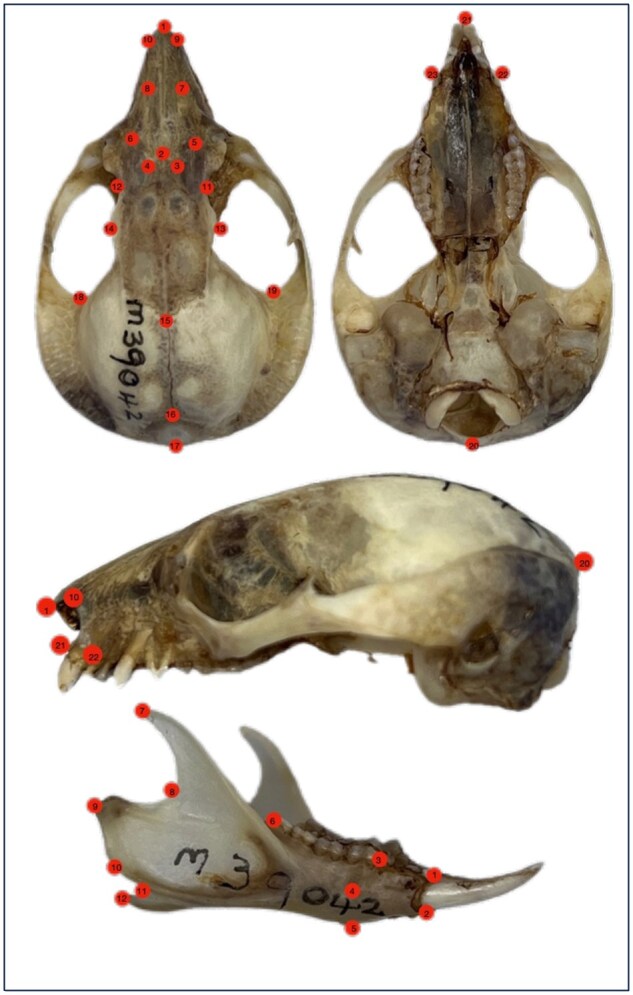
Position of landmarking points for cranium and mandible. Cranium on top left, right, and middle; and mandible in lower panel for all skulls.

Mandible and cranium specimens were adult, unbroken, and of known geographical origin within the research region. Landmarks were applied twice to 2D and 3D images to test for digitizing error (von Cramon-Taubadel et al. 2007). Following calculation of error and a review of digitization, digitizing error effect was rendered nonsignificant (Table 1, Fig. 1).

**Table 1.** Description of cranium (upper) and mandible (lower) landmarks used for geometric morphometric analysis.

Landmark number	Single or bilateral	*Cranium* landmark definitions and description (XYZ landmarks)
**1**	Single	Anteromedial point on the nasal
**2**	Single	Midsagittal point of the frontonasal suture
**3(R) & 4(L)**	Bilateral	Most dorsal point of the nasal (Right and Left)
**5(R) & 6(L)**	Bilateral	Intersection of the frontal, maxilla, and lacrimal suture (Right and Left)
**7 (R) & 8(L)**	Bilateral	Intersection of maxilla, nasal, and temporal suture (Right and Left)
**9(R) & 10(L)**	Bilateral	Anterior intersection of the nasal and maxilla suture (Right and Left)
**11(R) & 12(L)**	Bilateral	Most posterior point on the nasal (Right and Left)
**13(R) & 14(L)**	Bilateral	Post-orbital process of the frontal (Right and Left)
**15**	Single	Intersection of frontal and parietal suture
**16**	Single	Intersection of parietal and occipital suture
**17**	Single	Posterior most point on the sagittal crest
**18(L) & 19(R)**	Bilateral	Posterior point on temporal along zygomatic process
**20**	Single	Medial point on the dorsal border of the foramen magnum
**21**	Single	Anteromedial most point on the incisive
**22(L) & 19(R)**	Bilateral	Posterior border of the C1 alveolus

Landmark number	Single or bilateral	*Mandible* landmark definitions and description (XY landmarks)
**1**	Single	Intersection of the incisor’s superior posterior edge with the alveolar process
**2**	Single	Intersection of the incisor’s inferior posterior edge with the alveolar process
**3**	Single	Junction of posterior PM_1_ with the alveolar process
**4**	Single	Distal point of mental foramen
**5**	Single	Point at the maximum symphyseal height on body of mandible
**6**	Single	Junction of posterior M_3_ with the alveolar process
**7**	Single	Posterior-most point on the condyloid process’ articular surface
**8**	Single	Anterior-most point on the mandible’s caudal surface
**9**	Single	Posterior-most point on the angular process
**10**	Single	Posterior-most point on the angular process at the distal edge of the masseteric fossa
**11**	Single	Inferior point on the angular process distal to the masseteric fossa at the inversion
**12**	Single	Inferior-most point on the angular process

### Statistical analysis.

#### Data analysis.

A process of generalized Procrustes analysis was conducted, during which landmarked configurations were rescaled, translated, and rotated to obtain the Procrustes shape coordinates for all 2D and 3D images using MorphoJ v1.07a (Bookstein 1991, 1996; Klingenberg 2011).

#### Sexual dimorphism.

Male and female specimens were compared using Procrustes ANOVA for centroid size with sex. Significant differences were found between sexes for centroid size: all specimens, *F*(1,111) = 12.07, *P *= 0.007; Tasmania, *F*(1, 39) = 9.26, *P *= 0.0042; and Victoria, *F*(1, 70) = 3.28, *P *= 0.0746). All subsequent analyses treated sexes separately based on this preliminary finding.

#### Allometry

Using the shape coordinates for the cranium, an analysis of size on shape (allometry) was conducted. A regression analysis in MorphoJ was conducted using the Procrustes coordinates and centroid size to quantify the amount of shape variation that was influenced by centroid size (Savriama 2018). For all specimens, 5.6% of shape change was explained by allometry, and this subtle component of variation required no additional investigation.

#### Morphometric analysis.

Analyses were performed on the craniums and mandibles for specimens for each sex (females and males) between regions (Tasmania and Victoria). Principal Component Analysis (PCA) was performed to reduce the dimensionality of our landmarks data and identify components of variation expressed as percentages (Zelditch et al. 2012; Bookstein 2019). Differences between groups was assessed using a Procrustes ANOVA, testing the effect of group (Region) on shape variation for each sex. Using Discriminant Function Analysis (DFA), specimens were compared between Victoria and Tasmania to predict group membership between regions (Mitteroecker and Bookstein 2011). Canonical Variate Analysis (CVA) was used to visualize morphological variation between groups. Wireframe outlines, as a result of geometric morphometric methods and representing form for each region, were visually compared to investigate morphological variation (Klingenberg 2013).

## Results

**Fig. 2.**
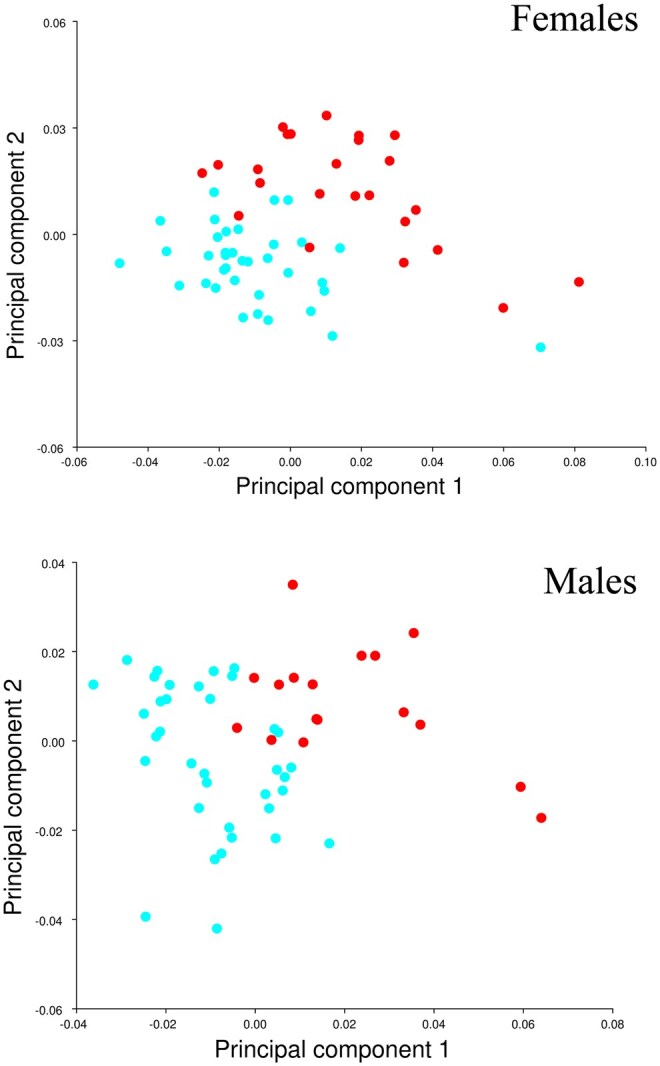
Principal Component Analysis results for females (above) and males (below). PC1 and PC2 compared showing clustering of Tasmanian specimens (red points) and Victorian (blue points).

**Fig. 3.**
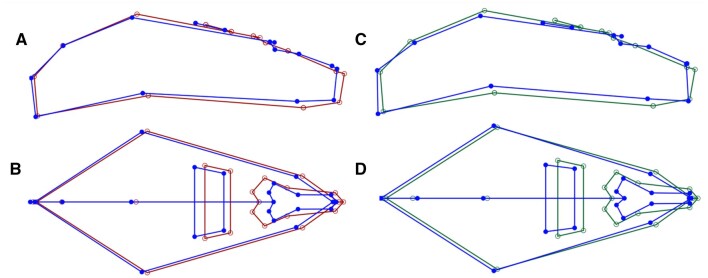
Wireframes showing the variance in PC1. Females (A lateral view, B dorsal view) and males (C lateral view, D dorsal view). The blue color shape is the changed shape (target) A and B (red) females starting shape and C and D (green) males starting shape.

The results of the PCA for the compared Tasmanian and Victorian populations showed that PC1 comprised 35.2% of the variation for females and 22.9% for males. The first 5 PCs in females and the first 7 in males represented >70% of the variation (Supplementary Data SD1 and SD2). Both the female and male PC1 and PC2 graphical comparison show clear clustering of each region (Fig. 2). Visual representation of the variation seen in the wireframes for PC1 show similar variation for both comparisons between sexes (Fig. 3).

### Cranium

Using Procrustes ANOVA on centroid size, a significant difference between Tasmanian and Victorian females (*F *= 5.79, *P *= 0.0193) was found. Landmark configuration analysis (shape) showed significant variation between the 2 regions (*F *= 4.32, *P *< 0.0001). For male craniums centroid size did not differ significantly between Tasmanian and Victorian individuals (*F *= 0.01, *P *= 0.9114). However, the landmark configurations (shape) differences were significant (*F *= 6.72, *P *< 0.0001).

Using the 3D coordinates of the cranium, significant variation was found in the DFA between specimens from Tasmania and Victoria. For females, the Procrustes distance between group means was 0.0346, and the Mahalanobis distance was 6.898. A Hotelling’s *T*^2^ test confirmed that this difference was highly significant (*T*^2^ = 685.25, *P *< 0.0001). Permutation tests based on 1,000 iterations also supported the significance of this result (Procrustes distance, *P *< 0.0001; *T*^2^, *P *< 0.0001). The DFA correctly classified 100% of individuals in the original sample (Tasmanian females, 24/24; Victorian females, 36/36). Cross-validation using the leave-one-out method showed a high classification accuracy of 90% overall, with 83.3% correct classification for Tasmanian specimens and 94.4% for Victorian specimens. For males, the Procrustes distance between group means was 0.0352, and the Mahalanobis distance was 8.417. A Hotelling’s *T*^2^ statistic confirmed that this difference was statistically significant (*T*^2^ = 818.07, *P *< 0.0001). Permutation tests with 1,000 iterations also supported the significance of both the Procrustes and Mahalanobis distances (*P *< 0.0001 for both). The DFA achieved 100% correct classification of individuals in the original dataset (Tasmanian males, 17/17; Victorian males, 36/36). Cross-validation using the leave-one-out method yielded a high classification accuracy of 87.3% overall, with 82.4% of Tasmanian males and 86.1% of Victorian males correctly classified.

**Fig. 4.**
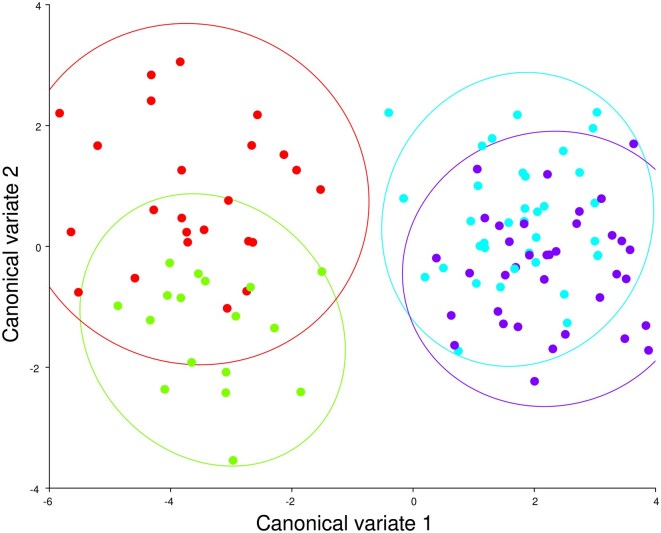
Canonical variate analysis for all specimens showing CV1 and CV2. Tasmanian female specimens represented by red points and male specimens represented by green points. Victorian female specimens represented by blue points and male specimens represented by purple points. 95% confidence ellipses represented in color for each sex and region. Tasmanian specimens clustering on left and Victorian specimens clustering on right.

Canonical Variate Analysis (CVA) indicated significant cranial shape differences among sex and geographic groups. The first 3 canonical variates explained 100% of the total variation (CV1 = 88.97%, CV2 = 7.03%, CV3 = 4.00%). A global permutation test strongly supported differences among group means (Goodall’s *F *= 6.76, *P *< 0.0001; Pillai’s trace = 1.49, *P *< 0.0001). Mahalanobis distances between groups were largest between Tasmanian and Victorian individuals of the same sex (e.g., T.F vs. V.F = 5.47, *P *< 0.0001; T.M vs. V.M = 5.75, *P *= 0.0001), while within-location sex differences (e.g., T.F vs. T.M = 2.42, *P *= 0.6386) were not significant. Victorian male and female groups were also not significantly different (*P *= 0.3042). Procrustes distances followed a similar pattern. The greatest distances occurred between Tasmanian and Victorian individuals of the same sex (T.F vs. V.F = 0.0346, *P *< 0.0001; T.M vs. V.M = 0.0352, *P *< 0.0001). Within-location sex comparisons showed no significant difference (T.F vs. T.M = 0.0180, *P *= 0.1064; V.F vs. V.M = 0.0119, *P *= 0.1074). These results confirm substantial geographic shape differentiation in cranial morphology, with weaker or nonsignificant effects of sex within each region (Fig. 4).

### Mandible

Procrustes ANOVA revealed significant shape differences between Tasmanian and Victorian individuals for both female and male mandibles. For females, shape variation between regions was highly significant (*F *= 18.24, *P *< 0.0001; Pillai’s trace = 0.84, *P *< 0.0001), although differences in centroid size were not statistically significant (*F *= 3.25, *P *= 0.0752). In contrast, male mandibles showed significant differences in both shape (*F *= 19.08, *P *< 0.0001; Pillai’s trace = 0.91, *P *< 0.0001) and centroid size (*F *= 7.84, *P *= 0.0071), indicating significant geographic variation in both.

**Fig. 5.**
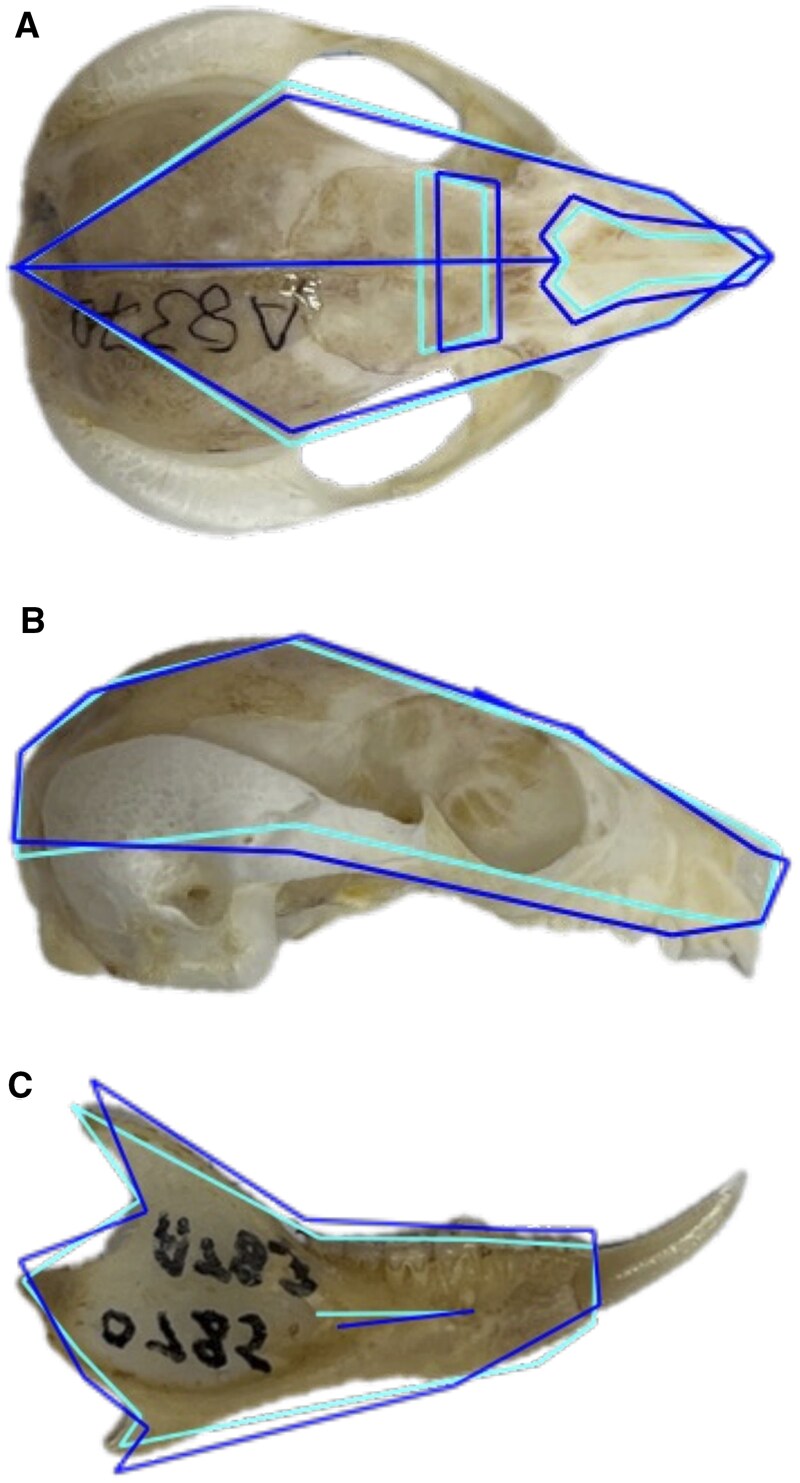
Wireframes representing shape changes between female Tasmanian and Victorian specimen averages. (A) Dorsal view cranium, (B) lateral view cranium, and (C) labial view mandible. Light blue line represents average shape for Tasmanian population and dark blue represents average shape for Victorian population.

**Fig. 6.**
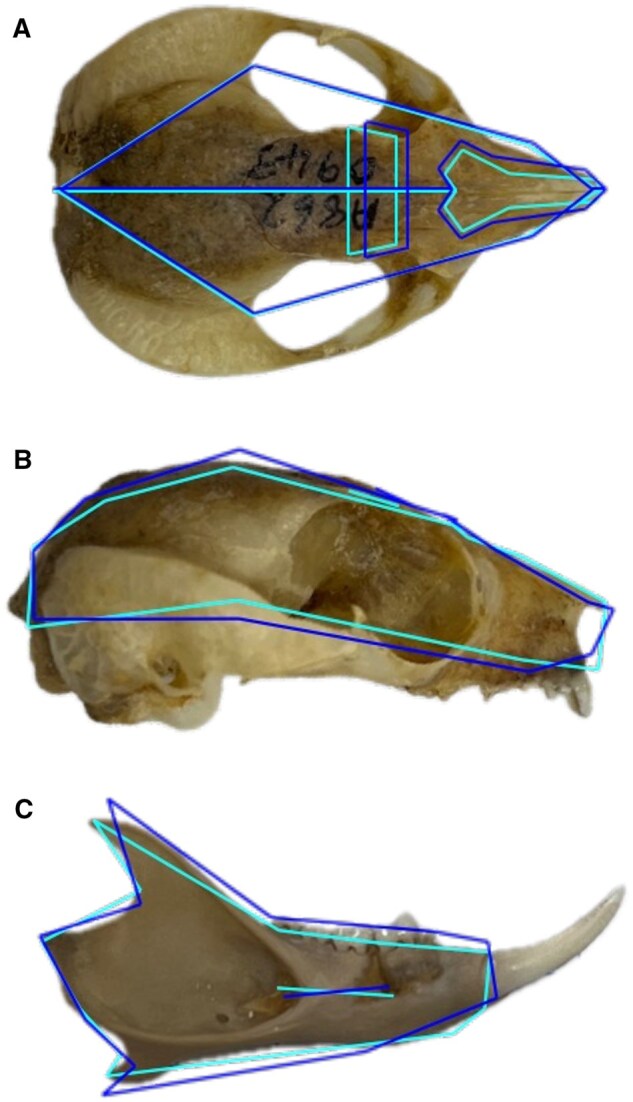
Wireframes representing shape changes between male Tasmanian and Victorian specimen averages. (A) Dorsal view cranium, (B) lateral view cranium, and (C) labial view mandible. Light blue line represents average shape for Tasmanian population and dark blue represents average shape for Victorian population.

Using DFA for females, the analysis found a statistically significant difference in mandible shape between the groups. The Procrustes distance between group means was 0.0469, and the Mahalanobis distance was 4.467. A Hotelling’s *T*^2^ statistic confirmed the significance of this difference (*T*^2^ = 395.13, *P *< 0.0001). Results from 1,000-permutation tests also confirmed significance for both Procrustes and Mahalanobis distances (*P *< 0.0001 for both), reinforcing strong group-level differentiation. The original DFA correctly classified 98.8% of individuals, with 100% of Tasmanian females (36/36) and 97.7% of Victorian females (43/44) correctly assigned. Cross-validation using the leave-one-out method yielded 95.8% overall classification accuracy, with 100% of Tasmanian females and 90.9% of Victorian females correctly classified. For males, Procrustes distance between group means was 0.0608, and the Mahalanobis distance was 6.786. The Hotelling’s *T*^2^ test confirmed the significance of this difference (*T*^2^ = 567.11, *P *< 0.0001). Permutation testing (1,000 iterations) further supported these results, with *P *< 0.0001 for both the Procrustes distance and *T*^2^ statistic. The DFA achieved 100% classification accuracy in the original dataset (Tasmanian, 18/18; Victorian, 39/39). Cross-validation using the leave-one-out method showed similarly high performance, with 97.9% overall classification accuracy; all Tasmanian males were correctly classified (100%), and 38 out of 39 Victorian males (97.4%) were correctly identified. Using the wireframes generated as a result of the Procrustes superimposition—where images are centered, scaled, and orientation are removed—an average shape for each location and sex were produced (Tasmanian and Victorian) for both the cranium and the mandible (Figs 5 and 6).

Craniums specimens from Tasmania were wider at the zygomatic/temporal region, with narrowed nasal bones and posteriorly positioned frontal bones when compared to the Victorian specimens. The insertion positions for the incisors and the canine teeth were the same height along the maxilla for the Tasmanian specimens. The Victorian specimens had a shorter nasal bone to incisor insertion height anteriorly and subsequently the maxilla canine and incisor tooth insertion locations were not aligned on the rostrum (as seen in the Tasmanian craniums). The Victorian incisor projection was angled forward when biting when compared to the more vertical bite for the Tasmanian specimens. Cranium height was reduced in the Tasmanian specimens especially anteriorly; however, the cranium was more developed and rounded posteriorly. The Tasmanian mandible body is narrower; however, there was an anteriorly located widening below the premolar region. The coronoid process of the Tasmanian mandible was shorter, narrower and more posteriorly angled. The angular process and overall size of the mandible body of the Tasmanian population was smaller than the Victorian. The masseteric fossa extended lower in the mandible body for the Victorian specimens.

## Discussion

Success for a translocated species is associated with fundamental events including transportation, introduction, establishment, and spread (Williamson 1996). For *P. notatus* in Tasmania, transportation as pets, subsequent escape, local establishment, and spread across Tasmania is an example of a translocated species enduring and adapting to a novel environment. Spreading and maintaining their population without anthropogenic support was likely associated with their non-herbivorous diet, small body mass, producing more than 1 offspring, and the large ranges of climatically suitable habitat (Forsyth et al. 2004; Latham et al. 2017). Our research investigated the skull form of introduced Tasmanian *P. notatus* and compared them with specimens from within their endemic geographic distribution and founding region of Victoria. We hypothesized that as an introduced species associated with identified predation behaviors of nesting bird species, it is likely that morphological variation would be identified between the Tasmanian and Victorian specimens. We found significant differences in the skull forms of the specimens from the 2 locations. The results show that *P. notatus* skulls in Tasmania are longer and wider with other indications of morphological variation when compared to the Victorian skulls.

Fauna in their endemic range naturally exhibit trait variation among conspecifics (Bolnick et al. 2011; Moran et al. 2016). When species are translocated to a novel environment, previous ecological constraints may be removed, thereby enabling morphological and behavioral divergence from the founding population. Variation identified in introduced species form and differing from founding phenotypes is well documented (Geist 1989; Bolnick et al. 2011; González-Suárez et al. 2015). Tasmanian *P. notatus* skull variation is likely associated with multiple contributing factors including low propagule pressure, climatic variables, and phenotype plasticity (Blackburn and Duncan 2001; Ghalambor et al. 2007; Tabak et al. 2018; Stobo-Wilson et al. 2020; Stringham and Lockwood 2021). Low propagule pressure (introduction effort) in Tasmania for *P. notatus* has previously been identified in low levels genetic diversity among specimens (Campbell et al. 2018). Approximately 5 individuals were estimated to contribute to the genetic diversity currently found in Tasmania for *P. notatus*. This effect may have contributed to phenotypic variation found in the Tasmanian gliders. Phenotype plasticity in an introduced species allows the traits that are required for sustaining the population to move closer to the phenotypic optimum for the new environment. Whether these traits are adaptive and specifically induced to suit the new environment, or represent a non-adaptive response to stress, the differences observed in the Tasmanian specimens may nevertheless have contributed to their success in that environment (Geist 1989; Ghalambor et al. 2007).

The comparison of the skull form from the 2 regions found characteristics in the Tasmanian average that may be associated with an increase in carnivory. The overall increase in size of the skull and particularly in the rostrum provides a wider gape consistent with Australian marsupial carnivores (extant *Antechinus* spp., *Dasyurus* spp., and extinct *Thylacine* spp.). An elongation of the rostrum allows for high velocity activity at the canines; a behavior associated with predation on agile prey items (Attard et al. 2011). A larger head would also allow for management of larger prey (Chemisquy et al. 2021; Mitchell et al. 2024; Warburton et al. 2024). A dorsal shift of the frontal bones was observed in the Tasmanian gliders is also seen in other carnivores (Tseng and Flynn 2018). The observed dorsal shift was a result of the overall widening and lengthening of the maxilla and specifically in the premolar region of the maxilla. The elongation of the premolar region and dorsal angulation of the coronoid process could also indicate a dietary shift towards carnivory (Prevosti et al. 2012; Grossnickle 2020).

Recent reports indicate that some bird species in Tasmania, particularly summer migratory species for breeding, have been impacted by predation from *P. notatus* (Stojanovic et al. 2014; Heinsohn et al. 2015; Stojanovic et al. 2017). Prior to the introduction of *P. notatus* in Tasmania, interaction between gliders and migratory or endemic nesting birds would not have occurred. The success of *P. notatus* in Tasmania may be associated with an available dietary niche for the predator and prey size. Of the other Tasmania marsupial carnivores, *D. viverrinus* (Eastern Quoll), whose diet includes mammal and bird species (Jones 1995), could overlap with identified bird predation by *P. notatus*. However, only the female Eastern Quoll has been identified as consuming small prey with a mass of 42 g or less. Male eastern quolls and other carnivores in Tasmania consume prey with masses > 154 g, likely beyond the capacity of a glider (Jones 1997). Furthermore, distribution and abundance of *D viverrinus* has decreased significantly since European settlement in Tasmania (Fancourt et al. 2013), likely reducing predator pressure by *D viverrinus* on bird species. The agility and smaller body size of *P. notatus* when compared with other Tasmanian carnivores likely facilitates access to hollow-nesting birds out of reach of other arboreal predators. A native bird species adaptive response has not been identified against this introduced predator species (Berthon 2015). An available dietary niche for *P. notatus*, with few other predators accessing small prey items, may be a reason for the success of gliders in Tasmania (Roughgarden 1972).

One limitation of our study was using specimens from Tasmania and Victoria, excluding other regions where *P. notatus* are also located. Further analysis may provide regional size and shape context for *P. notatus* throughout their Australian distribution. There were no specimens in museum collections prior to 1915 (*n *= 2)—most other specimens were collected from 1960 onwards, potentially missing historical evolutionary size and shape evidence of variation. Our size and shape analysis could be supported with a dietary study of *P. notatus* in Tasmania to provide an analysis of extent of bird inclusion as a dietary item. Live captures of additional specimens could also contribute to the assessment of body size between populations to further test our findings.

## Supplementary Material

gyaf074_Supplementary_Data

## Data Availability

Data for this research may be available by request to authors after publication.

## Supplementary data

**Supplementary Data SD1.**  Table S1. Female Victorian and Tasmanian 3D coordinate data PCA results with eigenvalues, the percentage of variance for each PCA and a cumulative percentage for 100% of variation.

**Supplementary Data SD2.**  Table S2. Male Victorian and Tasmanian 3D coordinate data PCA results with eigenvalues, the percentage of variance for each PCA and a cumulative percentage for 100% of variation.
