# Supplementary material for: Phenotype response for the invasive Petaurus notatus in Tasmania
Source: J Mammal. 2025 Oct 27;106(6):1456–64. doi: 10.1093/jmammal/gyaf074 (PMC12704938; doi:10.1093/jmammal/gyaf074)
Supplement: gyaf074_Supplementary_Data [file gyaf074_supplementary_data.zip › Appendix 1.docx]

| \| **Female**  **SPECIMEN no.** \| **STATE** \| \| --- \| --- \| \| QVM:1990:1:0009 \| Tasmania \| \| QVM:1988:1:0008 \| Tasmania \| \| QVM_2006_1_0038 \| Tasmania \| \| QVM_1990_1_11 \| Tasmania \| \| QVM:1986:1:0034 \| Tasmania \| \| QVM_1984_1_229 \| Tasmania \| \| QVM_1987_1_1 \| Tasmania \| \| QVM:1984:1:0246 \| Tasmania \| \| QVM:1987:1:0093 \| Tasmania \| \| QVM:2015:1:0028 \| Tasmania \| \| QVM_2015_1_0027 \| Tasmania \| \| QVM_1987_1_32 \| Tasmania \| \| QVM:1963:1:0210 \| Tasmania \| \| QVM:1988:1:0038 \| Tasmania \| \| QVM:1983:1:0041 \| Tasmania \| \| QVM:1965:1:0153 \| Tasmania \| \| QVM:1963:1:0300 \| Tasmania \| \| A1306 \| Tasmania \| \| A426 \| Tasmania \| \| A1417 \| Tasmania \| \| A1520 \| Tasmania \| \| A1471 \| Tasmania \| \| A1261 \| Tasmania \| \| A783 \| Tasmania \| \| A749 \| Tasmania \| \| A1511 \| Tasmania \| \| QVM:1984:1:261 \| Tasmania \| \| QVM_1987_1_25 \| Tasmania \| \| SA7307 \| Tasmania \| \| A1416 \| Tasmania \| | \| **Female**  **SPECIMEN no.** \| **STATE** \| \| \| \| --- \| --- \| --- \| --- \| \| C5765 \| \| Victoria \| \| C22455 \| \| Victoria \| \| C22457 \| \| Victoria \| \| C31702-1 \| \| Victoria \| \| C19120 \| \| Victoria \| \| C38163 \| \| Victoria \| \| C38165 \| \| Victoria \| \| C11589 \| \| Victoria \| \| C22460-1 \| \| Victoria \| \| C19118 \| \| Victoria \| \| C29890 \| \| Victoria \| \| C8734 \| \| Victoria \| \| C3007 \| \| Victoria \| \| C22649 \| \| Victoria \| \| C4285-1 \| \| Victoria \| \| C19133 \| \| Victoria \| \| C26612 \| \| Victoria \| \| C19009 \| \| Victoria \| \| C5567 \| \| Victoria \| \| C4839 \| \| Victoria \| \| C27626 \| \| Victoria \| \| C29763-1 \| \| Victoria \| \| C18877 \| \| Victoria \| \| C3012 \| \| Victoria \| \| C19212 \| \| Victoria \| \| C33042-1 \| \| Victoria \| \| C19121 \| \| Victoria \| \| C26038 \| \| Victoria \| \| C25274 \| \| Victoria \| \| C25896 \| \| Victoria \| \| C2783 \| \| Victoria \| \| C22474 \| \| Victoria \| \| C19207 \| \| Victoria \| \| C19132 \| \| Victoria \| \| C19126 \| \| Victoria \| \| C2146 \| \| Victoria \| \| C7777 \| \| Victoria \| \| C26479 \| \| Victoria \| \| C29789 \| \| Victoria \| \| C26027 \| \| Victoria \| \| C36800 \| \| Victoria \| \| C40096-1 \| \| Victoria \| \| C4320 \| \| Victoria \| \| C35911 \| \| Victoria \| \| C22475 \| \| Victoria \| \| C19010 \| \| Victoria \| \| C5766 \| \| Victoria \| \| C28104 \| \| Victoria \| \| C22461 \| \| Victoria \| \| C29755-1 \| \| Victoria \| \| C39848-1 \| \| Victoria \| \| C29761-1 \| \| Victoria \| \| C8176 \| \| Victoria \| \| C19205 \| \| Victoria \| \|  \| \|  \| |
| --- | --- | --- | --- | --- | --- | --- | --- | --- | --- | --- | --- | --- | --- | --- | --- | --- | --- | --- | --- | --- | --- | --- | --- | --- | --- | --- | --- | --- | --- | --- | --- | --- | --- | --- | --- | --- | --- | --- | --- | --- | --- | --- | --- | --- | --- | --- | --- | --- | --- | --- | --- | --- | --- | --- | --- | --- | --- | --- | --- | --- | --- | --- | --- | --- | --- | --- | --- | --- | --- | --- | --- | --- | --- | --- | --- | --- | --- | --- | --- | --- | --- | --- | --- | --- | --- | --- | --- | --- | --- | --- | --- | --- | --- | --- | --- | --- | --- | --- | --- | --- | --- | --- | --- | --- | --- | --- | --- | --- | --- | --- | --- | --- | --- | --- | --- | --- | --- | --- | --- | --- | --- | --- | --- | --- | --- | --- | --- | --- | --- | --- | --- | --- | --- | --- | --- | --- | --- | --- | --- | --- | --- | --- | --- | --- | --- | --- | --- | --- | --- | --- | --- | --- | --- | --- | --- | --- | --- | --- | --- | --- | --- | --- | --- | --- | --- | --- | --- | --- | --- | --- | --- | --- | --- | --- | --- | --- | --- | --- | --- | --- | --- | --- | --- | --- | --- | --- | --- | --- | --- | --- | --- | --- | --- | --- | --- | --- | --- | --- | --- | --- | --- | --- | --- | --- | --- | --- | --- | --- | --- | --- | --- | --- | --- | --- | --- | --- | --- | --- | --- | --- | --- | --- | --- | --- | --- | --- | --- | --- | --- | --- | --- | --- |
| \| **Male**  **SPECIMEN no.** \| **STATE** \| \| --- \| --- \| \| **SPECIMEN** \| **state** \| \| QVM:1987:1:0073 \| Tasmania \| \| QVM:1963:1:0297 \| Tasmania \| \| QVM:1986:1:0013 \| Tasmania \| \| A4913 \| Tasmania \| \| QVM:1963:1:0111 \| Tasmania \| \| QVM:1965:1:0152 \| Tasmania \| \| A430 \| Tasmania \| \| A1304 \| Tasmania \| \| QVM_1987_1_31 \| Tasmania \| \| QVM:1963:1:0039 \| Tasmania \| \| QVM:1987:1:0043 \| Tasmania \| \| QVM_1986_1_53 \| Tasmania \| \| QVM:1987:1:0027 \| Tasmania \| \| A1497 \| Tasmania \| \| A1508 \| Tasmania \| \| A8524 \| Tasmania \| \| A1502 \| Tasmania \| \| A1407 \| Tasmania \| \| A494 \| Tasmania \| \| A1403 \| Tasmania \| \| A1515 \| Tasmania \| \| A1429 \| Tasmania \| \| A923 \| Tasmania \| \| QVM_1985_1_1 \| Tasmania \| \| QVM_1984_1_242 \| Tasmania \| \| QVM_1987_1_50 \| Tasmania \| \| QVM_2014_1_27 \| Tasmania \| \| QVM_1984_1_263 \| Tasmania \| \| SA7306 \| Tasmania \| | \| **Male**  **SPECIMEN no.** \| **STATE** \| \| \| \| --- \| --- \| --- \| --- \| \| C38097 \| \| Victoria \| \| C19119 \| \| Victoria \| \| C10116-1 \| \| Victoria \| \| C2550 \| \| Victoria \| \| C960-1 \| \| Victoria \| \| C10869 \| \| Victoria \| \| C19127 \| \| Victoria \| \| C27725 \| \| Victoria \| \| C29690 \| \| Victoria \| \| C959-1 \| \| Victoria \| \| C8735 \| \| Victoria \| \| C18874 \| \| Victoria \| \| C31205 \| \| Victoria \| \| C31701-1 \| \| Victoria \| \| C27025 \| \| Victoria \| \| C22482 \| \| Victoria \| \| C3006 \| \| Victoria \| \| C3010-1 \| \| Victoria \| \| C33027 \| \| Victoria \| \| C41148-1 \| \| Victoria \| \| C31366 \| \| Victoria \| \| C29762 \| \| Victoria \| \| C2310-1 \| \| Victoria \| \| C3011 \| \| Victoria \| \| C35022-1 \| \| Victoria \| \| C5568 \| \| Victoria \| \| C19134 \| \| Victoria \| \| C31747 \| \| Victoria \| \| C19122 \| \| Victoria \| \| C19124 \| \| Victoria \| \| C38328-1 \| \| Victoria \| \| C37332-1 \| \| Victoria \| \| C2863 \| \| Victoria \| \| C22671 \| \| Victoria \| \| C35950-2 \| \| Victoria \| \| C28396 \| \| Victoria \| \| C27627 \| \| Victoria \| \| C22476 \| \| Victoria \| \| C22459 \| \| Victoria \| \| C2638-1 \| \| Victoria \| \| C38161-1 \| \| Victoria \| \| C41224-1 \| \| Victoria \| \| C41222 \| \| Victoria \| \| C26676 \| \| Victoria \| \| C18875 \| \| Victoria \| \| C9085 \| \| Victoria \| \| C19208 \| \| Victoria \| \| C19125 \| \| Victoria \| \|  \| \|  \| |
