# Supplementary material for: Phenotype response for the invasive Petaurus notatus in Tasmania
Source: J Mammal. 2025 Oct 27;106(6):1456–64. doi: 10.1093/jmammal/gyaf074 (PMC12704938; doi:10.1093/jmammal/gyaf074)
Supplement: gyaf074_Supplementary_Data [file gyaf074_supplementary_data.zip › Supplementary_Data_SD1.docx]

|  | Eigenvalues | % Variance | Cumulative % |
| --- | --- | --- | --- |
| 1. | 0.00013233 | 35.167 | 35.167 |
| 2. | 0.00005906 | 15.694 | 50.860 |
| 3. | 0.00003179 | 8.447 | 59.307 |
| 4. | 0.00002163 | 5.748 | 65.055 |
| 5. | 0.00002024 | 5.377 | 70.432 |
| 6. | 0.00001481 | 3.936 | 74.368 |
| 7. | 0.00001392 | 3.699 | 78.068 |
| 8. | 0.00001205 | 3.203 | 81.270 |
| 9. | 0.00000991 | 2.632 | 83.903 |
| 10. | 0.00000892 | 2.370 | 86.273 |
| 11. | 0.00000811 | 2.156 | 88.429 |
| 12. | 0.00000692 | 1.839 | 90.268 |
| 13. | 0.00000632 | 1.680 | 91.948 |
| 14. | 0.00000451 | 1.198 | 93.147 |
| 15. | 0.00000424 | 1.126 | 94.273 |
| 16. | 0.00000351 | 0.932 | 95.205 |
| 17. | 0.00000329 | 0.874 | 96.079 |
| 18. | 0.00000283 | 0.753 | 96.832 |
| 19. | 0.00000234 | 0.622 | 97.453 |
| 20. | 0.00000216 | 0.575 | 98.028 |
| 21. | 0.00000164 | 0.436 | 98.463 |
| 22. | 0.00000155 | 0.411 | 98.874 |
| 23. | 0.00000127 | 0.338 | 99.213 |
| 24. | 0.00000105 | 0.279 | 99.492 |
| 25. | 0.00000082 | 0.218 | 99.710 |
| 26. | 0.00000053 | 0.141 | 99.851 |
| 27. | 0.00000033 | 0.087 | 99.938 |
| 28. | 0.00000023 | 0.062 | 100.000 |

Supplementary Data SD1 Table 1. Female Victorian and Tasmanian 3D coordinate data PCA results with eigen values, the percentage of variance for each PCA and a cumulative percentage for 100% of variation.
