# Supplementary material for: Phenotype response for the invasive Petaurus notatus in Tasmania
Source: J Mammal. 2025 Oct 27;106(6):1456–64. doi: 10.1093/jmammal/gyaf074 (PMC12704938; doi:10.1093/jmammal/gyaf074)
Supplement: gyaf074_Supplementary_Data [file gyaf074_supplementary_data.zip › Supplementary_Data_SD2.docx]

Supplementary Data SD2 Table 2. Male Victorian and Tasmanian PCA results with eigen values, the percentage of variance for each PCA and a cumulative percentage for 100% of variation.

|  | Eigenvalues | % Variance | Cumulative % |
| --- | --- | --- | --- |
| 1. | 0.00043116 | 21.856 | 21.856 |
| 2. | 0.00026142 | 13.252 | 35.108 |
| 3. | 0.00019912 | 10.094 | 45.201 |
| 4. | 0.00017987 | 9.118 | 54.319 |
| 5. | 0.00014475 | 7.338 | 61.657 |
| 6. | 0.00010327 | 5.235 | 66.892 |
| 7. | 0.00009291 | 4.709 | 71.602 |
| 8. | 0.00008317 | 4.216 | 75.818 |
| 9. | 0.00007825 | 3.967 | 79.784 |
| 10. | 0.00006617 | 3.354 | 83.139 |
| 11. | 0.00004632 | 2.348 | 85.487 |
| 12. | 0.00003949 | 2.002 | 87.488 |
| 13. | 0.00003738 | 1.895 | 89.383 |
| 14. | 0.00003253 | 1.649 | 91.032 |
| 15. | 0.00002689 | 1.363 | 92.395 |
| 16. | 0.00002561 | 1.298 | 93.694 |
| 17. | 0.00002120 | 1.075 | 94.768 |
| 18. | 0.00001792 | 0.908 | 95.677 |
| 19. | 0.00001351 | 0.685 | 96.361 |
| 20. | 0.00001213 | 0.615 | 96.976 |
| 21. | 0.00001110 | 0.563 | 97.539 |
| 22. | 0.00000986 | 0.500 | 98.039 |
| 23. | 0.00000794 | 0.402 | 98.442 |
| 24. | 0.00000755 | 0.383 | 98.824 |
| 25. | 0.00000610 | 0.309 | 99.133 |
| 26. | 0.00000525 | 0.266 | 99.399 |
| 27. | 0.00000303 | 0.153 | 99.553 |
| 28. | 0.00000279 | 0.141 | 99.694 |
| 29. | 0.00000206 | 0.104 | 99.798 |
| 30. | 0.00000171 | 0.087 | 99.885 |
| 31. | 0.00000120 | 0.061 | 99.946 |
| 32. | 0.00000060 | 0.030 | 99.977 |
| 33. | 0.00000037 | 0.019 | 99.995 |
| 34. | 0.00000009 | 0.005 | 100.000 |
